# Prevention of the exposure by cyclophosphamide oral tablet

**DOI:** 10.1186/s40780-015-0020-9

**Published:** 2015-07-16

**Authors:** Takae Hanada, Yoichiro Takami, Kei Moriyama, Masafumi Oro, Takehiro Ogawa, Hiroko Moriyasu, Yuka Inoue, Asako Kanemitsu, Eiko Kawamoto, Ayaka Nagase, Anna Hamahara, Atsuko Yamamoto, Kenichi Shimada, Masashi Takahashi, Takashi Egawa

**Affiliations:** School of Pharmacy, Shujitsu University, 1-6-1 Nishigawara, Naka-ku, Okayama, 703-8516 Japan; Department of Pediatrics, Okayama University Hospital, 2-5-1 Shikata-cho, Kita-ku, Okayama, 700-8558 Japan; Muscat Pharmacy, 1290-1 Tamasu, Kita-ku, Okayama, 701-1154 Japan

**Keywords:** Cyclophosphamide, Oral tablet, Blister pack

## Abstract

**Background:**

Unintended exposure to antitumor agents from an oral medicine may place healthcare workers and patients taking medicine at risk. In this study, the exposure to blister pack by CP (cyclophosphamide) and appropriate preventive procedures were examined.

**Findings:**

CP detected inside the blister pack of the tested seven lots by LC-MS/MS ranged from 8.2 to 199.6 ng. Raman imaging clearly showed that CP ingredient was completely covered by the tablet coating layer and had not leached out of the tablet. In addition, the amount of CP detected inside the vials was suppressed under the lower detection limit until day 28, and only 6.0 ng was detected only at day 56.

**Conclusions:**

Various amounts of CP were contaminated to not only the inside of the blister pack but also the outside. This contamination may be caused not only by the manufacturing environment but also by the CP oral tablets themselves through volatilization of CP. Refrigerated storage of CP oral tablets may protect healthcare workers and patients from contact with CP.

**Electronic supplementary material:**

The online version of this article (doi:10.1186/s40780-015-0020-9) contains supplementary material, which is available to authorized users.

## Background

Although antitumor agents are very widely used for the treatment of cancer, they pose a risk to healthcare workers who handle the drugs or work in their vicinity and in patients, even at very low concentration. Exposure by antitumor agents to healthcare workers has been extensively studied over the last two decades. Antitumor agents have been detected in urine, blood and skin from healthcare workers and also in their work environment [1–3]. To prevent the exposure by antitumor agents, country-specific guidelines have been established and closed system devices have been used [4]. However, exposure of healthcare workers to antitumor agents is still observed.

Cyclophosphamide (CP), one of volatile antitumor agents, is also one of the most frequently used drugs. Exposure to CP in various healthcare working environments has been found with contamination of working trays, floors, surfaces inside biological safety cabinets, door handles, and injection vials [5, 6]. CP contamination on the surface of injection vials has been well examined; however, possible contamination on blister packs has been only rarely investigated [7]. The exposure by antitumor agents from oral medicine may place the healthcare workers and patients taking the medicine at risk. Here we evaluated possible exposure to CP on blister packs and determined ways to prevent CP exposure.

## Materials and methods

### Chemicals and materials

CP oral tablets (Endoxan® tablets) were obtained from Shionogi Co. Ltd. Cyclophosphamide monohydrate as a standard CP was obtained from Wako (Osaka, Japan). Hexamethylphosphoramide (HMPA) as an internal standard was obtained from ALDRICH (St Louis, USA). All other chemicals and solvents were of the highest analytical grades available.

### Analytical procedure

Chromatographic separation was performed using High-performance liquid chromatography system (Agilent 1100 series; Agilent, USA) and a CAPCELL PAK C18 MGII S-5 (100 mm × 2.0 mm; i.d., 3 μm; SHISEIDO, Japan) analytical column at 40 °C. The isocratic mobile phase consisted of mobile phase-A (0.1 % formic acid) and mobile phase-B (acetonitrile) (50:50, v/v) at a flow rate of 0.2 mL/min. The solution was filtered using a 0.22 μm membrane.

Mass spectrometric detection was performed on an ABSciex API 2000 triple quadrupole mass spectrometer (ABSciex, Toronto, Canada). Data acquisition was performed using the Analyst™ 1.6.1 software (AB Sciex, Toronto, Canada). The mass spectrometer was operated in the positive ion mode. Optimized instrument settings specific atropine and IS were as follows: curtain gas was 30 psi, ion source gas 1 was 70 psi, ion source gas 2 was 80 psi, ionspray voltage was 4000 V, and turbo heater temperature was 300 °C. Quantification was performed in the multiple reaction monitoring (MRM) mode with mass-to-charge (m/z) transitions at 261.2 > 140.1 for CP and 180.2 > 135.1 for IS.

### Preparation of the standard solution and quality control samples

Stock solutions of both CP monohydrate and HMPA were prepared at 1 mg/mL in methanol. The stock solutions of CP were diluted to 5, 10, 20, 50, 100 and 200 ng/mL in water containing 0.05 % formic acid and 50 % acetonitrile in water. HMPA stock solutions were diluted and added to both standard and sample solutions at the final concentration of 30 ng/mL.

### Sample preparation

**Fig. 1:**
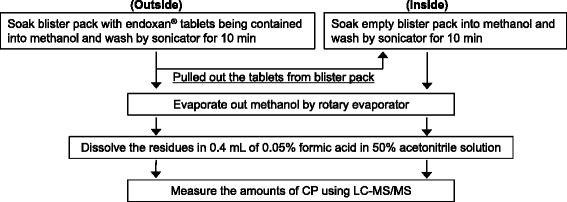
Schematic representation of sample preparation procedure prior to measurement of cyclophosphamide (CP) attached to the inside or outside of a blister pack separately

To measure the amounts of CP attached inside and outside the blister pack separately, the blister pack was washed twice. First, the blister pack containing the tablets was washed by methanol with 10 min of sonication. Next, the CP oral tablets were taken off from the blister pack and residual empty blister pack was washed by methanol with 10 min sonication. These sample solutions were extracted and measured by LC-MS/MS (Fig. 1).

### Raman imaging

Detailed procedures for Raman imaging are described in Additional file 1.

## Results

**Fig. 2:**
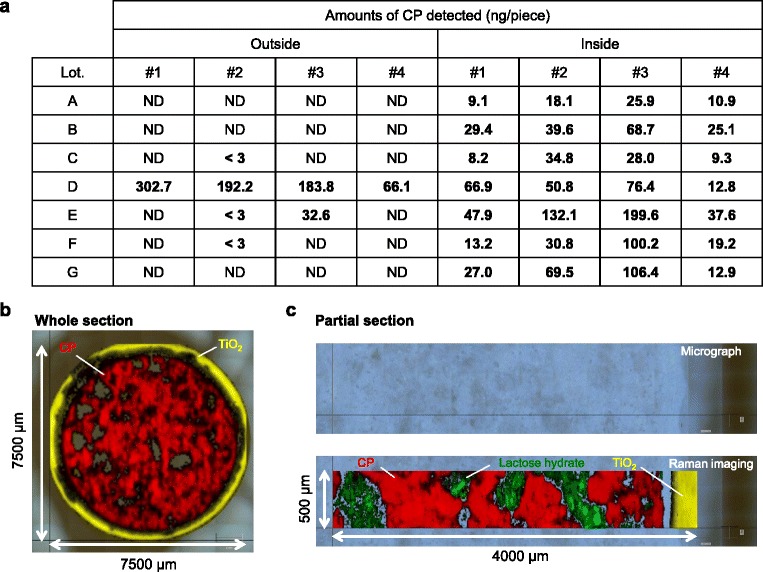
The amount of CP on the inside and outside of the blister packs, and localization of CP within the Endoxan® tablet. **a**. The amount of CP attached to the inside or outside of the blister pack was measured using liquid chromatography-tandem mass spectrometry (LC-MS/MS). #1, #2, #3 and #4 are replicate numbers assigned among the same lot. CP, cyclophosphamide; ND, not detected; < 3, below the detection limit (3 ng/sheet). **b** and **c**. Raman chemical imaging of (**b**) CP oral tablet whole and (**c**) a CP oral tablet cross-section. Red area, CP; Yellow area, titanium oxide; Green area, lactose hydrate

The amounts of CP attached to both the inside and outside of each blister pack were separately measured using LC-MS/MS. The CP extraction procedure is described in Fig. 1. As a result, CP ranged from 8.2 to 199.6 ng was detected inside the blister pack for all seven lots (Fig. 2a). In addition, a broad range of CP (~302.7 ng) was also detected outside the blister pack by some lots (Fig. 2a).

To clarify whether CP had leached out to the outside the tablet, CP localization at a whole tablet or tablet cross section was analyzed using a Raman imaging system. Interestingly, Raman imaging clearly demonstrated that the CP ingredient was completely covered by the coating layer containing titanium oxide, with no evidence of leaching (Fig. 2b and 2c).

Further, to examine whether the CP ingredient packed in tablet could volatilize to outside of the tablet and, if present, whether such CP volatilization could be suppressed by low temperature storage, 5 CP oral tablets were removed from a blister pack and the tablets were stored in a vial at either room temperature (RT) or 4 °C for 7, 14, 28 and 56 days. The average RT was 21.0 ± 1.7 (°C) during this experiment (see Additional file 2). Following storage, the CP attached to the inside of the vials was quantified using LC-MS/MS, respectively. From these analyses, CP was detected to be 8.0 ng inside a vial stored at RT for 7 days, with the amount of CP detected increasing significantly through day 56 (Table. 1). In addition, the amount of CP detected inside the vials was suppressed under the lower detection limit until day 28 and only 6.0 ng was detected at day 56.

**Table 1:** Sequential change of CP amounts attached to inside of stocked vials

			Stored periods			
			Day 7	Day 14	Day 28	Day 56
Amount of CP^a^ (ng/vial)	Vehicle	RT^b^	ND	ND	ND	ND
		4 °C	ND	ND	ND	ND
	Endoxan® tablets	RT	8.0 ± 1.2	10.3 ± 2.7	17.2 ± 2.0^*^	49.0 ± 6.7^**^
		4 °C	ND	<3	<3	6.0 ± 1.0^##^

^a)^ cyclophosphamide

^b)^ room temperature

Data are represented by mean ± SD. ^*^ *p* < 0.05; ^**^ *p* < 0.01, Dunnet’s multiple comparison test; ^##^ *p* < 0.01, unpaired *t*-test; ND; not detected, < 3; under the detection limit (3 ng/vial)

## Discussion

In this study, we demonstrated that various amounts of CP had contaminated both the inside and outside of the blister pack. There are many reports of CP contamination on the surface of CP injection vials and on their cap covers [7–9]. However, there is currently only one report of CP contamination on both the outside and inside of blister packs. Hedmer et al. reported that 0.5 ng median (range 0.2 − 3.5 ng) amounts of CP was detected on the outside of CP oral tablet blister packs (Sendoxan 50 mg tablets, Sweden) [7]. The authors have pointed out the need for a proper cleaning of manufacturing facilities and equipment to avoid such contamination. As presented in Fig. 2a, the amount of CP detected outside of the blister pack in Lot. D were unaccountably higher than those inside of the blister pack. In this experiment, all lots of blister packs were newly purchased. We ascertained all blister packs uncorrupted. In addition, samples for measurement and CP standard solutions were prepared on separate table by separate experimenters to avoid artificial contamination of CP. Thus, we think that outside of the blister packs may be randomely contaminated with volatiled CP from both the manufacturing environment and from the CP oral tablets themselves. We evaluated the localization of CP in whole tablet and tablet cross sections using a Raman imaging system to clarify whether CP had leached out of the tablet. However, tablet’s CP ingredient was completely covered by the coating layer and had not leached outside the tablet (Fig. 2b and 2c). Thus, CP was thought to have been volatilized from the tablet.

Furthermore, we showed that such CP contamination through volatilization could be prevented by refrigerated storage (Table. 1). Endoxan® tablets are generally recommended for storage at room temperature. Our results showed that CP contamination inside the blister pack could be suppressed below the detection limit for at least 28 days by refrigeration (Table. 1). CP oral tablets are often prepared as a one-dose package in Japan and are not prescribed for more than one month because of efficacy monitoring and the occurrence of adverse effects in treated patients. In fact, the most frequently prescribed period was 28 days from Jan 2015 to Mar 2015 at Muscat Pharmacy (see Additional file 3). Thus, the refrigerated storage of CP oral tablets may protect healthcare workers and patients from unintended contact with the hazardous agents, CP.

## Findings

We demonstrated that various amounts of CP had contaminated both the inside and the outside of the blister pack. This contamination could be because of both the manufacturing environment and the CP oral tablets themselves through volatilization. We suggest refrigerated storage of CP oral tablets to protect healthcare workers and patients from unintended contact CP exposure.

## Additional files

Additional file 1:
**Supplemental Procedure and Refferences.**
Additional file 2:
**Supplementary Table.**
Additional file 3:
**Supplementary Table.**

## Abbreviations

CP: Cyclophosphamide
HMPA: Hexamethylphosphoramide
LC-MS/MS: Liquid Chromatography-tandem mass spectrometry
HPLC: High-performance liquid chromatography
