# Supplementary material for: Prevention of the exposure by cyclophosphamide oral tablet
Source: J Pharm Health Care Sci. 2015 Jul 16;1:20. doi: 10.1186/s40780-015-0020-9 (PMC4728781; doi:10.1186/s40780-015-0020-9)
Supplement: Additional file 1: — Supplemental Procedure and Refferences. [file 40780_2015_20_MOESM1_ESM.docx]

**Additional File. 1 - Supplemental Procedure and Refferences**

**Raman imaging of whole tablet CP oral tablet or tablet cross section**

The single-point Raman spectra of materials used in this study were recorded on an inVia Raman microscope system (Renishaw plc., Gloucestershire, UK) equipped with a Leica microscope and a 785 nm, 300 mW excitation laser. CP oral tablet was fixed on a glass plate with an instant glue and milled with a milling machine (Proxxon GmbH, Niersbach, Germany) to expose the cross-section. Raman chemical imaging of the tablet cross-section was performed on an inVia Raman microscope system using Renishaw StreamLine^TM^ Plus high-speed Raman line-mapping technology under a 785 nm excitation laser [1-3]. For whole section imaging, a 7500 μm ×7500 μm area including whole area of the cross-section was applied to imaging at a spatial resolution of 56.7 μm (17956 data points in total) using a 5× objective lens. For partial section imaging, a 3800 μm × 500 μm area, extending from an edge to the center of the tablet cross-section, was analyzed at a 7.1 μm spatial resolution (38664 data points in total) using a 20× objective lens. Data collection and analyses were performed using Wire 4.1 software (Renishaw plc.). Discrimination of materials was performed according to direct classical least squares modelling using the single-point Raman spectrum of each component as indices [4].

**Supplemental Refeneces**

1 Sasic S, Clark DA, Mitchell JC, Snowden MJ: Raman line mapping as a fast method for analyzing pharmaceutical bead formulations. Analyst. 2005;130: 1530-6.

2 Sasic S and Clark DA. Defining a strategy for chemical imaging of industrial pharmaceutical samples on raman line-mapping and global illumination instruments. Applied spectroscopy. 2006;60:494-502.

3 Henson MJ and Zhang L. Drug characterization in low dosage pharmaceutical tablets using raman microscopic mapping. Applied spectroscopy. 2006;60: 1247-55.

4 Vajna B, Farkas I, Szabó A, Zsigmond Z, Marosi G. Raman microscopic evaluation of technology dependent structural differences in tablets containing imipramine model drug. J Pharm Biomed Anal. 2010;51: 30-8.
