# Supplementary material for: Prevention of the exposure by cyclophosphamide oral tablet
Source: J Pharm Health Care Sci. 2015 Jul 16;1:20. doi: 10.1186/s40780-015-0020-9 (PMC4728781; doi:10.1186/s40780-015-0020-9)
Supplement: Additional file 3: — Supplementary Table. [file 40780_2015_20_MOESM3_ESM.docx]

**Additional File. 3 – Supplementary Table**

**The frequency of prescribed period for CP oral tablet from Jan 2015 to Mar 2015 at Muscat Pharmacy**

| Patient | Date (prepared) | | Daily dose (tablet) | Prescribed period (days) |
| --- | --- | --- | --- | --- |
| A | 10-Feb-15 | 1 | | 6 |
|  | 17-Feb-15 | 1 | | 6 |
|  | 23-Feb-15 | 1 | | 7 |
|  | 03-Mar-15 | 1 | | 7 |
|  | 16-Mar-15 | 1 | | 7 |
| B | 29-Jan-15 | 1 | | 7 |
|  | 26-Feb-15 | 1 | | 7 |
| C | 29-Jan-15 | 1 | | 7 |
| D | 21-Jan-15 | 1 | | 7 |
|  | 10-Feb-15 | 1 | | 7 |
| E | 10-Mar-15 | 1 | | 7 |
| F | 21-Jan-15 | 1 | | 7 |
|  | 13-Jan-15 | 0.5 | | 8 |
|  | 10-Feb-15 | 0.5 | | 8 |
|  | 17-Mar-15 | 0.5 | | 14 |
| G | 08-Jan-15 | 1 | | 14* |
|  | 05-Mar-15 | 1 | | 14* |
|  | 13-Mar-15 | 1 | | 14* |
|  | 27-Mar-15 | 1 | | 14* |
| H | 14-Jan-15 | 1 | | 17 |
|  | 28-Jan-15 | 1 | | 21 |
|  | 18-Feb-15 | 1 | | 21 |
|  | 25-Mar-15 | 1 | | 21 |
| I | 30-Jan-15 | 1 | | 22 |
|  | 27-Feb-15 | 1 | | 22 |
|  | 27-Mar-15 | 1 | | 28 |
| J | 16-Jan-15 | 2 | | 28* |
|  | 20-Feb-15 | 2 | | 28* |
|  | 20-Mar-15 | 2 | | 28* |
| K | 15-Jan-15 | 1 | | 28* |
|  | 12-Feb-15 | 1 | | 28* |
|  | 12-Mar-15 | 1 | | 28* |
| L | 08-Jan-15 | 1 | | 28 |
|  | 12-Mar-15 | 1 | | 28 |
| M | 27-Feb-15 | 1 | | 28 |
| N | 07-Jan-15 | 1 | | 28 |
|  | 14-Jan-15 | 1 | | 28 |
|  | 21-Jan-15 | 1 | | 28 |
|  | 28-Jan-15 | 1 | | 28 |
|  | 18-Feb-15 | 1 | | 30 |
|  | 25-Feb-15 | 1 | | 30 |
|  | 04-Mar-15 | 1 | | 35 |
|  | 11-Mar-15 | 1 | | 35 |
|  | 25-Mar-15 | 1 | | 42 |
| O | 16-Jan-15 | 2 | | 56 |
|  | 30-Jan-15 | 2 | | 61 |
|  | 20-Feb-15 | 2 | | 63 |
|  | 20-Mar-15 | 2 | | 63 |

* prepared as a one dose package
